# Methotrexate-associated lymphoproliferative disease detected as a colorectal mass lesions: a case report

**DOI:** 10.1093/jscr/rjad098

**Published:** 2023-03-07

**Authors:** Katsudai Shirakabe, Ken Mizokami

**Affiliations:** Department of General Surgery, Tokyo Bay Urayasu Ichikawa Medical Center, Urayasu, Chiba, Japan; Department of General Surgery, Tokyo Bay Urayasu Ichikawa Medical Center, Urayasu, Chiba, Japan

**Keywords:** Methotrexate, Lymphoma, DLBCL, MTX-LPD

## Abstract

Methotrexate-related lymphoproliferative disorder (MTX-LPD) is a rare but serious complication that occurs in patients treated with methotrexate (MTX); although MTX-LPD has been reported recently, the incidence in the colon is very low. A 79-year-old woman who had been receiving MTX for 15 years came to our hospital complaining of postprandial abdominal pain and nausea. Computed tomography scan showed the dilation of the small bowel and a tumor in the cecum. In addition, numerous nodular lesions were seen in the peritoneum. Ileal-transverse colon bypass surgery was performed for small bowel obstruction. Histopathological findings of both the cecum and the peritoneal nodules revealed the diagnosis of MTX-LPD. We report MTX-LPD occurring in the colon; it is important to consider MTX-LPD when intestinal symptoms occur during MTX therapy.

## INTRODUCTION

Methotrexate (MTX) has generally been the first-line drug for RA treatment since its adoption in the 1980s; MTX has benefited a significant number of RA patients.

The first association between MTX and lymphoma was reported in 1991 when Ellman *et al*. found the development of lymphoma in RA patients treated with MTX. Since then, after similar cases were reported, the condition became known as MTX-LPD.

About 50% of MTX-LPD occurs in extralymphanode sites such as the lungs, skin and oropharynx, but it is uncommon to occur in the colon. About half of the MTX-LPD patients improve with the discontinuation of MTX.

Here, we report a rare case of small bowel obstruction because of MTX-LPD, in which an ileal-transverse colon bypass was performed.

## CASE REPORT

**Figure 1:**
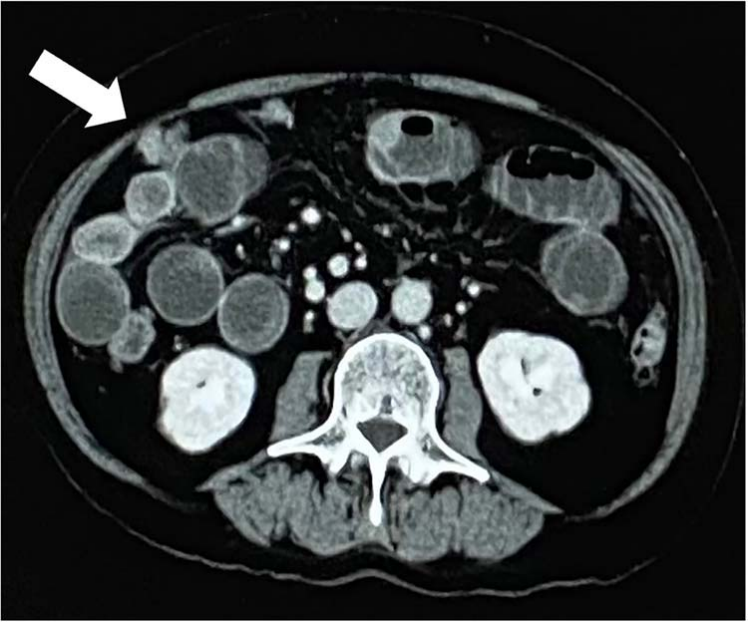
White arrow indicates peritoneal nodule.

**Figure 2:**
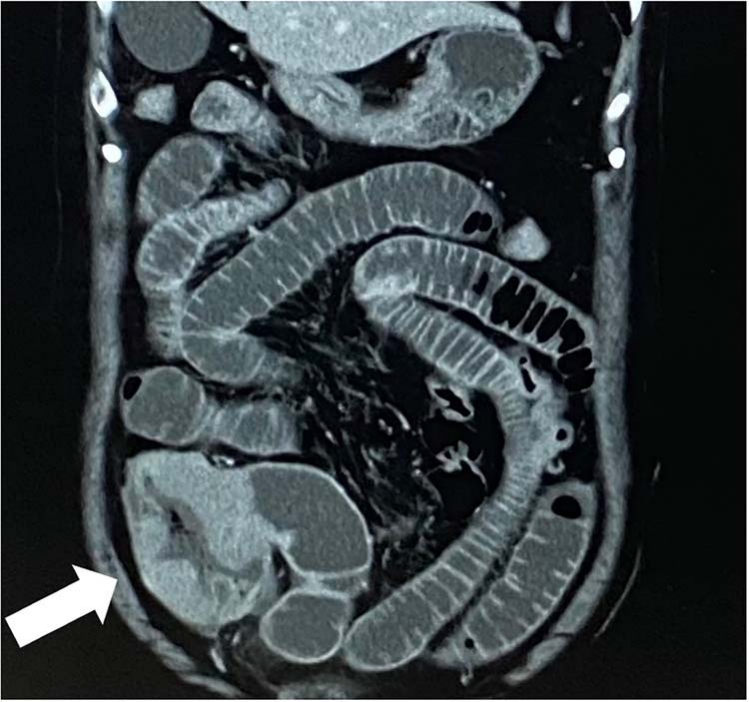
White arrow points to mass lesion in the cecum.

A 79-year-old woman, who had been taking MTX 4.0 mg/week for rheumatoid arthritis since she was 64 years old, came to our hospital complaining of vomiting repeatedly. Physical examination revealed no tenderness in the abdomen, but the abdomen was distended and a mass was observed in the right lower abdomen. Contrast-enhanced computed tomography revealed a mass lesion with contrast effect in the cecum and multiple peritoneal tumors (Figs 1 and 2).

**Figure 3:**
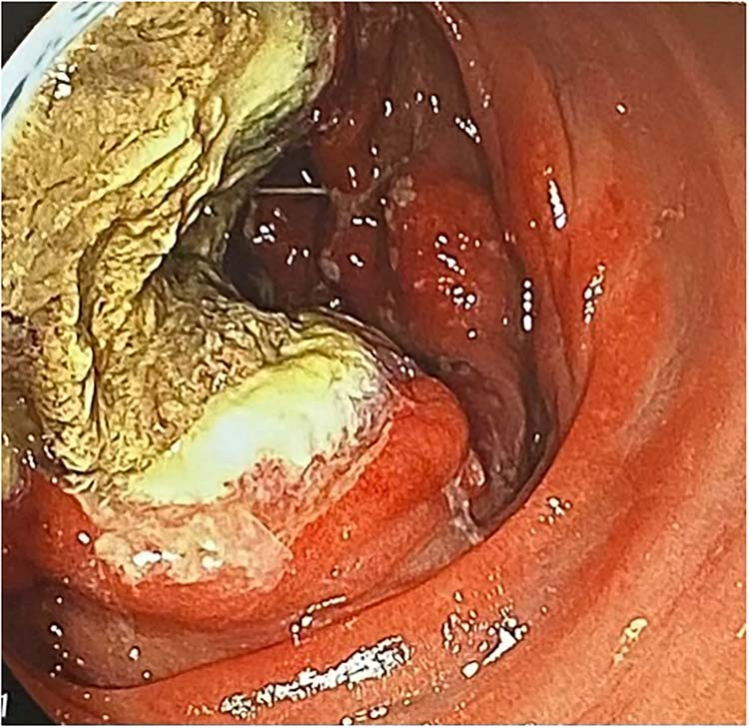
Colonoscopy revealed a mass lesion with ulcerative changes.

Colonoscopy revealed an irregular ulcerative lesion in the cecum, invading the Bauhin valve (Fig. 3).

Ileal-transverse colon bypass was performed because of the obstruction of the small intestine.

The patient underwent surgery after removal of the disseminated nodule for additional histopathological examination.

The postoperative course was unremarkable.

Immunohistochemistry showed CD5(+) and CD20(+) in both the ulcerative lesion in the cecum and the peritoneal disseminated nodule, and a diagnosis of diffuse large B cell lymphoma (DLCBL) was made.

Since the patient had been taking MTX, he was diagnosed with methotrexate-associated lymphoproliferative disorders (MTX-LPD) and MTX was discontinued.

The patient was transferred to another hospital for chemotherapy.

## DISCUSSION

MTX is known as an anticancer drug and immunosuppressive drug and is widely used as a treatment for rheumatoid arthritis in particular, and reports of MTX-LPD as a side effect of MTX have been increasing since Ellman *et al*. [1] reported.

Methotrexate-related lymphoproliferative disorder (MTX-LPD) is classified as ‘other iatrogenic immunodeficiency-related LPD’ in the World Health Organization’s *Classification of Tumors of Hematopoietic and Lymphoid Tissues*, Fourth Edition, similar to immunodeficiency-related LPD, including posttransplant LPD and human immunodeficiency-related LPD [2]. MTX causes various types of LPD, with DLCBL being the most common subtype, accounting for about half of the cases.

The etiology is still unknown, but histology has shown that it is primarily of B-cell origin, suggesting an association with EBV.

Pathologically, DLBCL is the most common, accounting for 35–60% of MTX-LPD cases, followed by Hodgkin lymphoma in 12–25% of cases [3].

Extralymphatic lesions are seen in about half of the cases, but the most common sites other than the lymph nodes are the lungs, skin and oropharynx, and cases affecting the large intestine are extremely rare [4].

Risk factors for the development of MTX-LPD include taking more than 8 mg/week [5], and RA with LPD patients report a significantly higher rate of EBV infection [3].

Diagnostic criteria for MTX-LPD have not established yet. The Japan College of Rheumatology recommends that the possibility of MTX-LPD be considered when RA patients on MTX therapy present with systemic symptoms such as indeterminate fever, general malaise and weight loss, or with laboratory abnormalities such as hepatosplenomegaly, abnormal white blood cell differential counts, decreased platelet counts, anemia and high lac-acid dehydrogenase [6]. Patients with lymphadenopathy or extralymphatic disease should be considered as well [7]; MTX-LPD is generally diagnosed based on both the pathology of the malignant lymphoma and history of MTX administration, with EBV positivity considered.

The first option for treatment is the discontinuation of MTX. Previous studies have reported that almost half of the MTX-LPD cases show regression with MTX discontinuation alone [3, 7].

Inui *et al*. [8] reported maximal tumor shrinkage only after 8 weeks of MTX discontinuation in 13 of 15 cases. Therefore, follow-up after more than 8 weeks is considered advisable to evaluate tumor response.

For these patients who do not respond to MTX withdrawal, conventional chemotherapy for lymphoma should be considered [3].

In this case, the patient was found as a colorectal mass and was suspected to have peritoneal dissemination.

It was accompanied by small bowel obstruction, and bypass surgery was performed. The histopathological diagnosis of the disseminated nodule was also a result of DLBCL.

It is important to keep in mind the possibility of MTX-LPD when a patient on MTX medication presents with gastrointestinal lesions.

Although MTX-LPD is rare, it has been increasing in recent years and care should be taken when treating it with MTX.

Treatment should include the discontinuation of MTX, which improves about half of the patients, and chemotherapy if there is no improvement after 8 or more weeks of discontinuation.

Patients being treated with MTX should be suspected of involvement if they present with lymphoma.
